# Enhanced Ion Current Rectification in 2D Graphene‐Based Nanofluidic Devices

**DOI:** 10.1002/advs.201500062

**Published:** 2015-05-08

**Authors:** Morteza Miansari, James R. Friend, Leslie Y. Yeo

**Affiliations:** ^1^ Department of Mechanical and Aerospace EngineeringMonash UniversityClaytonVIC3800Australia; ^2^ Micro/Nanophysics Research LaboratoryRMIT UniversityMelbourneVIC3001Australia

**Keywords:** asymmetry, graphene oxide, nanochannels, nanofluidics, rectification

## Abstract

Furthering the promise of graphene‐based planar nanofluidic devices as flexible, robust, low cost, and facile large‐scale alternatives to conventional nanochannels for ion transport, we show how the nonlinear current–voltage (*I*–*V*) characteristics and ion current rectification in these platforms can be enhanced by increasing the system asymmetry. Asymmetric cuts made to the 2D multilayered graphene oxide film, for example, introduces further asymmetry to that natively inherent in the structurally symmetric system, which was recently shown to be responsible for its rectification behavior due to diffusion boundary layer fore–aft asymmetry. Supported by good agreement with theory, we attribute the enhancement to the decrease in the limiting current in the positive bias state in which counter‐ion trapping occurs within the negatively charged graphene oxide sheets due to increased film permselectivity as its cross‐section and surface charge distribution is altered on one end; these effects being shown to be sensitive to the electrolyte pH. Further, we show that an imbalance in the pH or concentration in the microreservoirs flanking the film can also increase asymmetry and hence rectification, in addition to displaying a host of other phenomena associated with the *I*–*V* characteristics of typical nanochannel electrokinetic systems.

## 1 Introduction

In a similar way that the semiconductor diode is an indispensable building block in the electrical circuit toolbox, nanofluidic diodes^1^ which comprise synthetic nanopores and nanochannels that mimic the functions of voltage‐gated biological ion channels^2^, ^3^, ^4^, ^5^, ^6^ have now become a common component for ion/molecular transport and regulation in applications that span biosensing,^7^, ^8^, ^9^, ^10^ energy storage/conversion,^11^, ^12^, ^13^ and water purification,^14^, ^15^ amongst others. As with their electrical counterpart, the key to the function of the nanofluidic diode is its ability to rectify the current such that ion transport is enhanced in one direction and suppressed in another. Such ion current rectification is usually achieved through the introduction of asymmetry, whether in the nanopore/nanochannel geometry,^16^, ^17^, ^18^, ^19^, ^20^ its surface charge distribution,^21^, ^22^, ^23^, ^24^, ^25^, ^26^, ^27^ or in the properties of the electrolyte solution at both ends of the nanopore/nanochannel.^28^, ^29^

Recently, we observed that rectification is also displayed in ion transport across 2D carbon lattice (e.g., graphene‐based) materials, in particular, the “nanochannels” that make up the interstitial spaces between stacked sheets of graphene oxide (GO) films.^30^ In some ways, it was quite surprising that these GO films were observed to exhibit ion current rectification behavior, given their structural (or geometric) symmetry. This was attributed to the natural asymmetry in the ion transport through the films itself—the asymmetry arising from the peculiar susceptibility of the film to counter‐ion trapping and release within the gaps that make up the interstices between the stacked GO sheets. This ability to construct nanofluidic diodes simply from stacking GO films, which are quite easily synthesized and are fairly robust, thus represents an attractive large‐scale alternative to the costly and complex steps required in conventional nanopore and nanochannel fabrication.

In the present work, we seek to show how the rectification performance of these GO nanofluidic rectifiers can be additionally enhanced by further introducing fore–aft asymmetry to the structurally/geometrically symmetric system. While it may be intuitive that increasing the degree of asymmetry in the system through the means reported previously for nanopore/nanochannel systems (e.g., by incorporating geometrical asymmetry into the nanofluidic structure or by incorporating other asymmetric effects such as a nonuniform charge distribution along a geometrically symmetric nanofluidic structure) would lead to increased rectification, little is understood with regards to such effects in the GO film. We therefore report results from a systematic study on these effects and provide fundamental insight into our observations by elucidating the underlying physicochemical hydrodynamic mechanisms responsible for the phenomena. In addition, we also show, for the first time in 2D graphene‐based nanofluidic systems, the ability for electrical power generation by introducing a salinity difference across the GO film.

## 2 Results and Discussion

In structurally/geometrically symmetric GO films, it was recently found that ion current rectification arises—quite unexpectedly given the absence of any apparent asymmetry in the system—from counter‐ion trapping within the film during the positive bias cycle that retards ion migration through the negatively‐charged sheets, and their corresponding release during the negative bias cycle that leads to an enhanced electromigration flux through the sheets.^30^ It is this counter‐ion trapping and release that disrupts the electrokinetic fore–aft symmetry inherent in the structurally symmetric film, particularly in the diffusion boundary layer, thus endowing it with the rectification characteristics observed. It is also possible that the nonuniform electric field that arises within the sheets due to the tortuosity in the spaces between them also contributes to the inherent asymmetry of the system, although this appears to be a secondary effect. We show here that it is possible to increase the degree of rectification in the GO film by introducing further asymmetry to the system in a variety of ways, which we examine in turn: the film geometry (Section 3.1), solution pH (Section 3.2), and solution concentration (Section 3.3).

### 2.1 Geometrical Asymmetry

**Figure 1:**
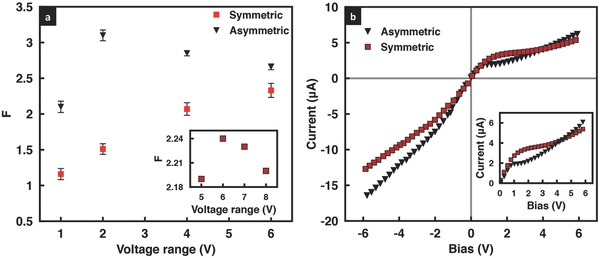
a) Rectification factor as a function of the voltage scan range, and b) corresponding *I*–*V* curves for a KCl solution concentration of 0.1 × 10^−3^ m at pH 7 for geometrically symmetric (red squares) and asymmetric (black triangles) GO films. The inset in (a) shows an extension to higher voltages for the symmetric case, indicating that a maximum in the rectification factor also arises in this case above a critical gating voltage of approximately 6 V, whereas the inset in b) is a magnification of the positive bias region, showing the earlier transition to the overlimiting region in the asymmetric case, as observed by the lower critical gating voltage of approximately 2 V.

**Figure 2:**
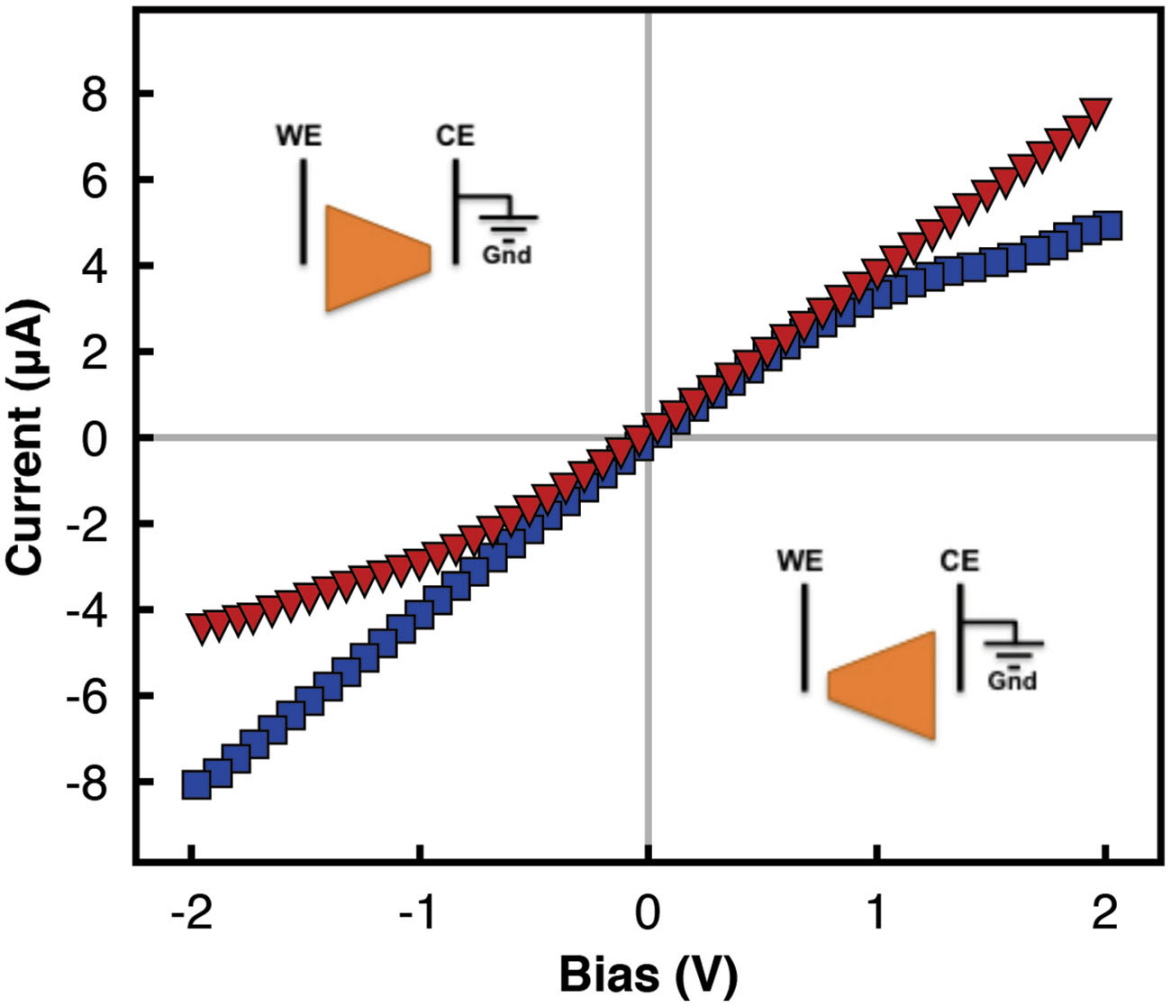
Effect of reversing the working and counter electrodes (WE and CE, respectively), with respect to the geometrically asymmetric film (cross‐sectional area ratio *b*/*a* = 3) on its *I*–*V* characteristics. The electrolyte concentration and pH was maintained at 0.1 × 10^−3^ m and a value of 7, respectively.

Reminiscent of symmetry breaking and rectification in conical nanopores,^1^, ^21^, ^31^, ^35^, ^36^ one way geometrical asymmetry can be introduced in a 2D planar structure such as the GO films examined here, for example, is to cut the film into a trapezoidal geometry such that one end is narrower than the other. **Figure** **1**a, in fact, shows that the rectification factor *F* (the ratio of the currents measured for applied voltages with the same amplitude but opposite polarities |*I*_(−*V*)_/*I*_(+*V*)_|, wherein *I*_(−*V*)_ and *I*_(+*V*)_ are the reverse and forward currents measured at negative and positive bias voltages applied to the working electrode (WE), respectively) approximately doubles with the tapered geometry (the dependence of *F* on the degree of tapering will be investigated subsequently), at least initially for voltages up to ±2 V. Beyond this voltage, however, we observe the rectification behavior of the asymmetric film to decrease despite the monotonic increase in the rectification with applied voltage in its symmetric counterpart, and hence the rectification enhancement to diminish considerably. As an aside, while swapping the working and counter electrodes with respect to the film did not lead to any differences for the geometrically symmetric film,^30^ we observe in contrast in **Figure** **2** that effects of the geometric asymmetry in the film (i.e., the saturation in the current at lower bias voltages and hence the lower limiting current values at the tapered end, the reason for which we elucidate below) are preserved, as would be expected. Nevertheless, we note that only the polarity of the current inverts; the magnitude of the current at a given voltage however remains similar, which is consistent with that shown previously for geometrically similar films wherein the rectification, and hence the underlying charge trapping mechanism responsible for giving rise to it, is insensitive to reversal of the electrodes.^30^

To understand the observations above, in particular, the maximum and subsequent decay in the rectification factor in the geometrically asymmetric film, we first examine the *I*–*V* characteristics of the system. As seen in Figure 1b, the *I*–*V* characteristics for these systems are essentially nonlinear and similar to that typically observed for nanopore/nanochannel geometries due to the ion permselectivity endowed in the pores as a consequence of the overlap in the Debye layers when their lengths become comparable to that of the nanopore/nanochannel dimension above a critical electrolyte concentration.^1^, ^32^, ^33^, ^34^ Briefly, the *I*–*V* curves for these systems comprise a linear Ohmic region at low voltages followed by a limiting current region above a threshold voltage over which the current plateaus when the ion concentration at the reservoir–film entrance vanishes. The current then starts to increase again beyond a critical gating voltage in what is known as the overlimiting current region due to the dominance of the electromigration flux over the diffusive flux in the nanopore/nanochannel, or in this case, the interstitial spaces between the GO sheets. The large current density that forms as a consequence of the convergence of the electric field from the micrometer dimension reservoir into the nanopore/nanochannel can only be sustained through the formation of an electroneutral diffusion layer at the reservoir–pore/channel entrance/exit in both the diffusion‐limited Ohmic and limiting current regions,^37^ whereas an additional concentration polarization layer known as the secondary double layer rich in counter‐ions forms in the overlimiting current region due to the strong electromigration flux.^38^, ^39^ From Figure 1b, it can be seen that both the symmetric and asymmetric GO films exhibit such typical nonlinear *I*–*V* characteristics although we note that the asymmetric film not only possesses a lower limiting current *I*_lim_ but also a shorter voltage range over which the limiting current behavior is observed, i.e., a lower critical gating voltage of around 2 V exists for the asymmetric film above which the current increases sharply (in contrast to the critical gating voltage of approximately 6 V for the symmetric case, as observed in Figure 1b, and the inset of Figure 1a); this is possibly due to the stronger electromigration flux in the film arising from the the increased intensity of the convective vortices in the secondary polarization layer^40^, ^41^ as a consequence of the field focusing effect at the narrow end of the asymmetric film. Given that the primary mechanism for the symmetry breaking and hence the observed rectification behavior in the GO film lies in the existence of the asymmetric diffusion boundary layers at both ends of the film as a consequence of the counter‐ion trapping and release,^30^ it then becomes clear that the disruption in the diffusion boundary layer to form the secondary double layer above the critical gating voltage of 2 V is the reason for the observed decrease in the rectification above this voltage in Figure 1a. A corresponding decrease in the rectification is not observed for the symmetric GO film in Figure 1a over the scan range of ±6 V because both its limiting current and critical gating voltage are considerably higher than its asymmetric counterpart, as seen in Figure 1b.

Here, we adopt, for simplicity, the 1D model by Yossifon et al.^37^ which captures the transport of ions through a nanochannel membrane flanked by two microreservoirs—a configuration that is similar to the present work given that the interstitial space between the sheets that make up the multilayered GO film of height *h* (≈1 nm after swelling in water^30^, ^42^) can be approximated by *n* discrete parallel “nanochannels” with effective height *h*_eff_ = *nh*. By matching the electric field and flux at the nanochannel–reservoir interfaces at both entrance and exit ends, Yossifon et al. were able to capture the electric field focusing effect that arises due to the convergence of the 2D field in the radially symmetric reservoir into the nanochannel, which is assumed to have a height that is much smaller than the reservoir dimension. The holistic treatment of the system, which not just considers the ion transport through the nanochannel, but also that in the bulk reservoir region as well as in the boundary layers at the nanochannel–reservoir interface, in addition to a departure from an equilibrium treatment of the Debye layer (i.e., the Donnan theory), allows a more comprehensive insight into the nonlinear *I*–*V* characteristics (particularly in the overlimiting region) in permselective nanoporous membranes. This is a particularly important consideration given that such nonlinear *I*–*V* characteristics and the associated diffusion boundary layer play a central role in the GO film and its rectification behavior that we explore here.^30^ Although the model does not capture the transient dynamics of the ion trapping and release within the GO sheets and hence the resulting asymmetry in the diffusion boundary layers, it provides a sufficiently adequate framework for interpreting our observations given that we simply seek insight into the limiting current behavior.

By solving the Nernst–Planck equations that govern the transport of ions of a symmetric electrolyte with equal diffusivities *D* together with Poisson's equation in each of the three regions together with appropriately matched interface and far‐field conditions, Yossifon et al. showed that it was possible to obtain the ionic flux entering the nanochannel and hence an expression for the limiting current.^32^, ^37^ Given our approximation of *n* nanochannels stacked in parallel to model the interstitial space between the sheets in the GO film of width *w* and length *L*, the limiting current across the film can simply be approximated by the sum of the limiting currents for each nanochannel in parallel, i.e.,

(1) $$I_{\lim}=\left(\frac{\eta +1}{\eta -1}\right)\frac{n\pi zFwDc_{0}}{\ln\;(h/Le)}$$

where *η* is the degree of permselectivity of the film with *η* → ∞ for ideal permselectivity. In the above, *c*_0_ is the concentration of the buffer solution in the bulk reservoir and *L*_*e*_ is the thickness of the concentration polarization layer that comprises the Debye layer, the diffusion boundary layer, and the secondary double layer.^43^ Yossifon et al. demonstrated that *L*_*e*_ can be adequately approximated by the distance between the nanochannel–reservoir interface and the electrode, which, in this case, is around 2 mm. We note from Equation (1) that the limiting current is dependent on both the width of the film *w* and hence its geometry at the entrance and exit, as well as the factor (*η* + 1)/(*η* − 1), which is related to the volume charge density in the film *X* = 2σ/*zFh*_eff_ (assumed uniform) through the ratio of the counter‐ion to co‐ion concentrations within the film, i.e., *η* = *c*_+_(*X*)/*c*_−_(*X*), assuming that the GO film is negatively charged. These, in turn, can be obtained from the equilibrium Donnan theory^44^

(2) $$c_{\pm }=\pm X/2+[{\left(X/2\right)}^{2}+c_{0}^{2}]1/2$$

We now proceed to use these relationships to understand the consequences of introducing geometrical asymmetry, in particular, through variations in the cross‐sectional area and the surface charge distribution across the GO film, on its *I*–*V* characteristics and rectification behavior.

**Figure 3:**
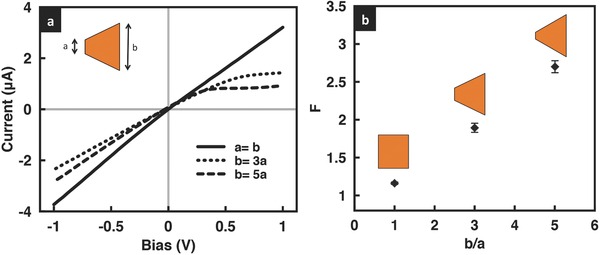
a) Representative *I*–*V* curves and b) rectification factors for GO films with different cross‐sectional areas at the entrance and exit ends for KCl solution concentrations of 0.1 × 10^−3^ m at pH 7.

**Figure 4:**
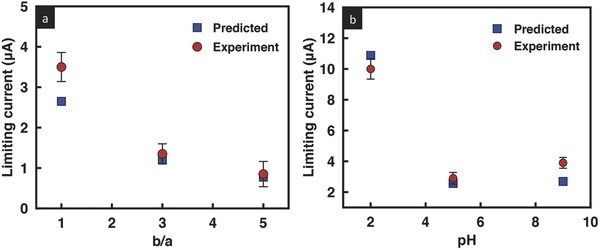
Comparison of the limiting current obtained experimentally (red circles) with that predicted theoretically (blue squares) for a) three different ratios of the film cross‐sectional area (the solution pH is held constant at 7), and b) at three different solution pH values for a given cross‐sectional area ratio *b*/*a* = 3. The concentration of the KCl solution is 0.1 × 10^−3^ m.

It can be seen from **Figure** **3** that the introduction of geometric fore–aft asymmetry by cutting the film leads to a departure from linearity in the film's *I*–*V* characteristics; the greater the asymmetry, captured through its cross‐sectional area ratio *b*/*a*, the more nonlinear the behavior that is observed. That this effect is only seen for positive bias voltage and not in the negative bias state, and thus the rectification that arises (the degree of rectification increasing with increasing asymmetry), was attributed to a push versus pull mechanism postulated by Miansari et al.^30^ In addition, we observe the limiting current in **Figures** 3a and **4**a to decrease with increasing geometric asymmetry, although we note that the decrease in the nonlinearity observed and in the limiting current appears to asymptote to a constant value as the film cross‐section at the entrance and exit ends becomes increasingly divergent.

This trend can also be seen in the prediction afforded by Equation (1). The surface charge density was obtained from the measured conductance across the film, which, for *n* parallel nanochannels, is simply *n* times the conductance of a single nanochannel, given by^29^, ^30^
(3)
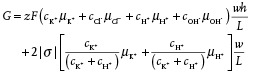
the first term on the right representing the bulk conductance, which dominates at high ionic strengths, and the second term the double layer conductance (including the fraction of excess K^+^ and H^+^ ions within the nanochannel), which dominates at low ionic strengths. In the above, $\mu_{K^{+}}=7.63\times 10^{-8}$ m^2^ V^−1^ s, $\mu_{{\mathrm{Cl}}^{‐}}=7.91\times 10^{-8}$ m^2^ V^−1^ s, $\mu_{H^{+}}=3.63\times 10^{-7}$ m^2^ V^−1^ s, and $\mu_{{\mathrm{OH}}^{‐}}=2.05\times 10^{-7}$ m^2^ V^−1^ s are the mobilities of the various ionic species in the system.^45^ Briefly, we first calculate *h*_eff_ by considering only the first term on the right side of Equation (3) and measuring the conductance at high salt concentration (1 m KCl) such that the film only exhibits bulk‐solution like conductance,^30^, ^42^ from which *n* = *h*_eff_/*h* can be estimated. We then obtain approximate values for σ by considering the full expression given in Equation (3) by measuring the total conductance at low ionic concentrations (0.1 × 10^−3^ m) and dividing by *n*. As seen from Figure 4a, relatively good agreement is obtained between the limiting current that is predicted with that observed experimentally. We note that although the limiting current is linearly dependent on the width of the film, as suggested by Equation (1), the effect of altering the cross‐sectional area of the film on the limiting current is much more complex given that *X* and hence *h*_eff_ varies for different geometries, thus leading to different values for (*η* + 1)/(*η* − 1).

**Figure 5:**
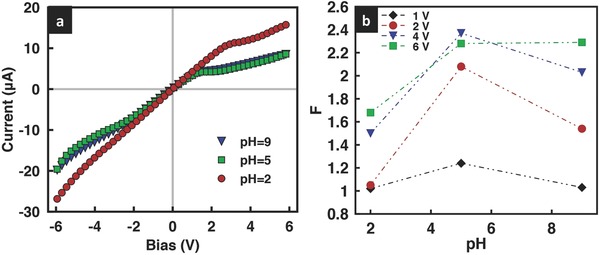
a) *I*–*V* characteristics of the geometrically asymmetric GO film (*b*/*a* = 3) for various electrolyte solution (0.1 × 10^−3^ m KCl) pH values; the working electrode WE is placed on the narrow side of the film. b) Corresponding rectification factor *F* as a function of pH.

The solution pH and hence the surface charge distribution within the film can also influence the limiting current in the geometrically asymmetric GO film (*b*/*a* = 3), as seen from Equation (1). To investigate this effect, we record the film's *I*–*V* characteristics for acidic (pH = 2 and pH = 5) as well as basic (pH = 9) solutions (**Figure** **5**), corresponding to estimated *η* values of approximately 1.44, 4.10, and 6.33, or (*η* + 1)/(*η* − 1) ≈ 5.54, 1.65, and 1.37, respectively. The increase in the film's permselectivity, reflected in the increase in the value of *η* with pH, is a consequence of the increase in the dissociation of the carboxylic acid groups in the GO sheets (COOH → COO^−^ + H^+^), which therefore leads to an increase in the number density of the COO^−^ surface charges.^30^ From Equation (1), we thus expect the *I*–*V* curves to become more nonlinear and hence the limiting current to decrease monotonically with increasing solution pH. However, it can be seen from both Figures 4b and 5 that the behavior is, in contrast, nonmonotonic. This is because the conductance did not significantly change with increasing pH above the dissociation point of the carboxyl groups along the surface of the GO sheets (pK_a_ ≈ 4.2), at which point the reduction in surface charge and the electrostatic repulsion associated with the overlapping Debye layers in the nanochannels result in the collapse of the film,^30^ thus reducing its height and cross‐sectional area in Equation (1), and therefore suppressing further decrease in the conductance and hence decrease in the limiting current at pH 5. In fact, we observe this effect to slightly overcompensate at high pH such that there is a small increase in the limiting current (Figure 4b) and corresponding decrease in the rectification factor (Figure 5b); the overcompensation becoming increasingly pronounced at higher voltages, consistent with our previously reported results for geometrically symmetric films.^30^ Parenthetically, we also note that Figure 5a suggests that the departure from nonlinearity can still be observed even if the nanochannels do not possess large ion‐selectivity at low pH values, although we observe the onset of nonlinearity at the limiting current to occur at larger currents and at higher applied voltages.

### 2.2 Solution pH Asymmetry

**Figure 6:**
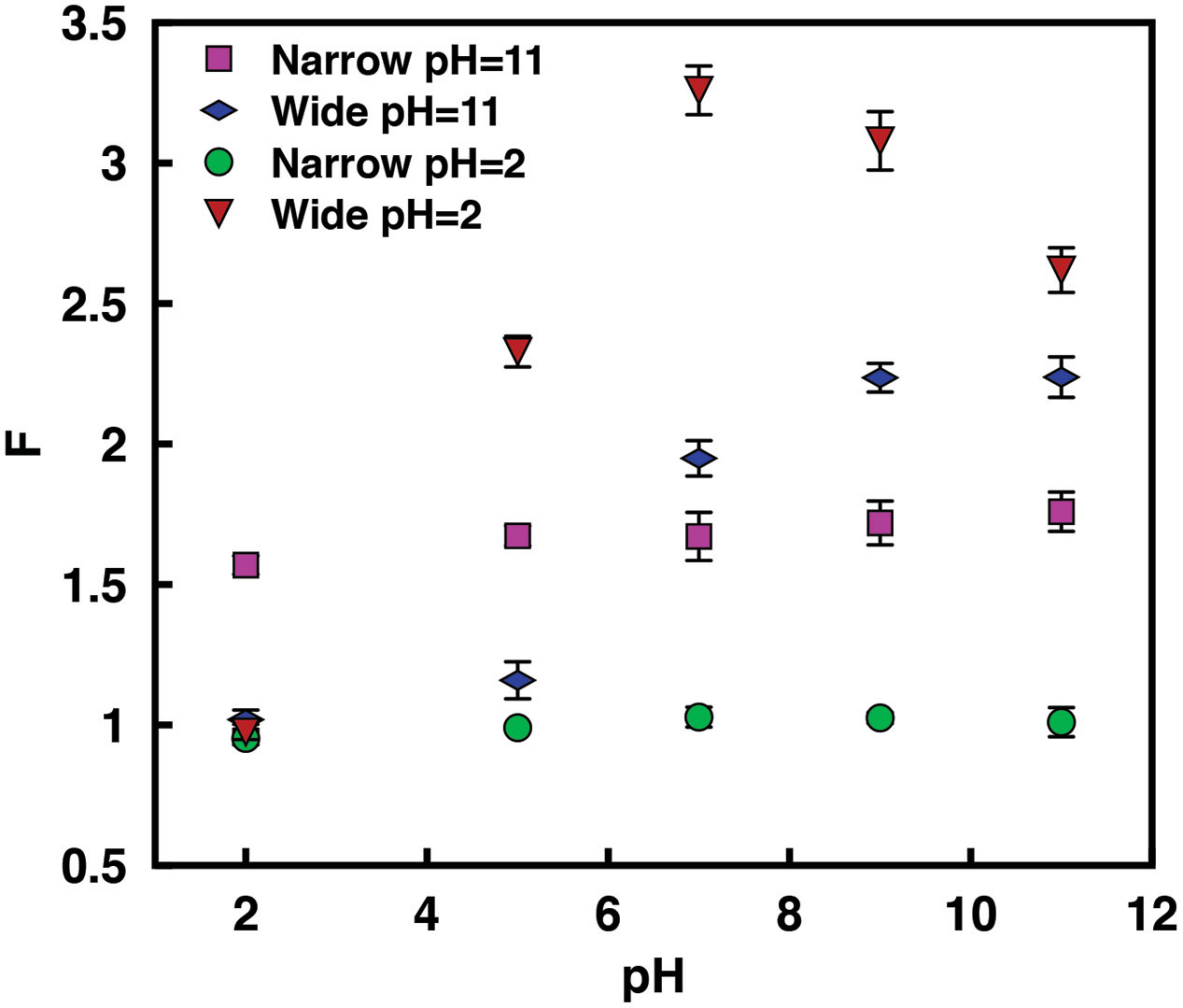
Rectification factor for the geometrically asymmetric GO film (*b*/*a* = 3) as the solution pH in the reservoir on either the cathode or anode end is varied while being held constant at the other. The concentration of the KCl solution is 0.1 × 10^−3^ m and the legend denotes the end at which the pH is held constant either at pH = 2 or pH = 11.

The above study examining the influence of the solution pH on the limiting current and hence the rectification behavior of GO films can be further extended by generating an asymmetric imbalance in the solution pH on the anodic and cathodic reservoirs surrounding a geometrically symmetric GO film. **Figure** **6** shows the degree of rectification to be very sensitive to variations in the solution pH in the reservoir that interfaces the narrow end (while being held constant at the wide end) compared to the case when the solution pH is varied in the reservoir at the wide end (and held constant at the narrow end), which is almost insensitive to the pH variation. This is because of the compounding effects of both the cross‐sectional area and the surface charge distribution on the limiting current previously discussed in Section 3.1. More specifically, the rectification ratio *F* is observed to continuously increase when the pH at the wide end is held at highly basic conditions (pH = 11) and the pH at the narrow end is gradually increased until the pH levels are symmetric across the film. We postulate that this is a result of the decrease in *I*_(+*V*)_ due to the opposing cation (K^+^) diffusion from the high pH end to the low pH end of the GO film. In contrast, *F* is observed to decrease again above pH 7 at the narrow end when the pH at the wide end is held under acidic conditions (pH = 2) due to a reversal in the direction of the cation diffusion, thus leading to a decrease in *I*_(−*V*)_ and hence the rectification ratio (Figure 6). Below, we will isolate these confounding effects to allow us to examine the effects of asymmetry in the solution pH alone by considering a geometrically symmetric GO film.

**Figure 7:**
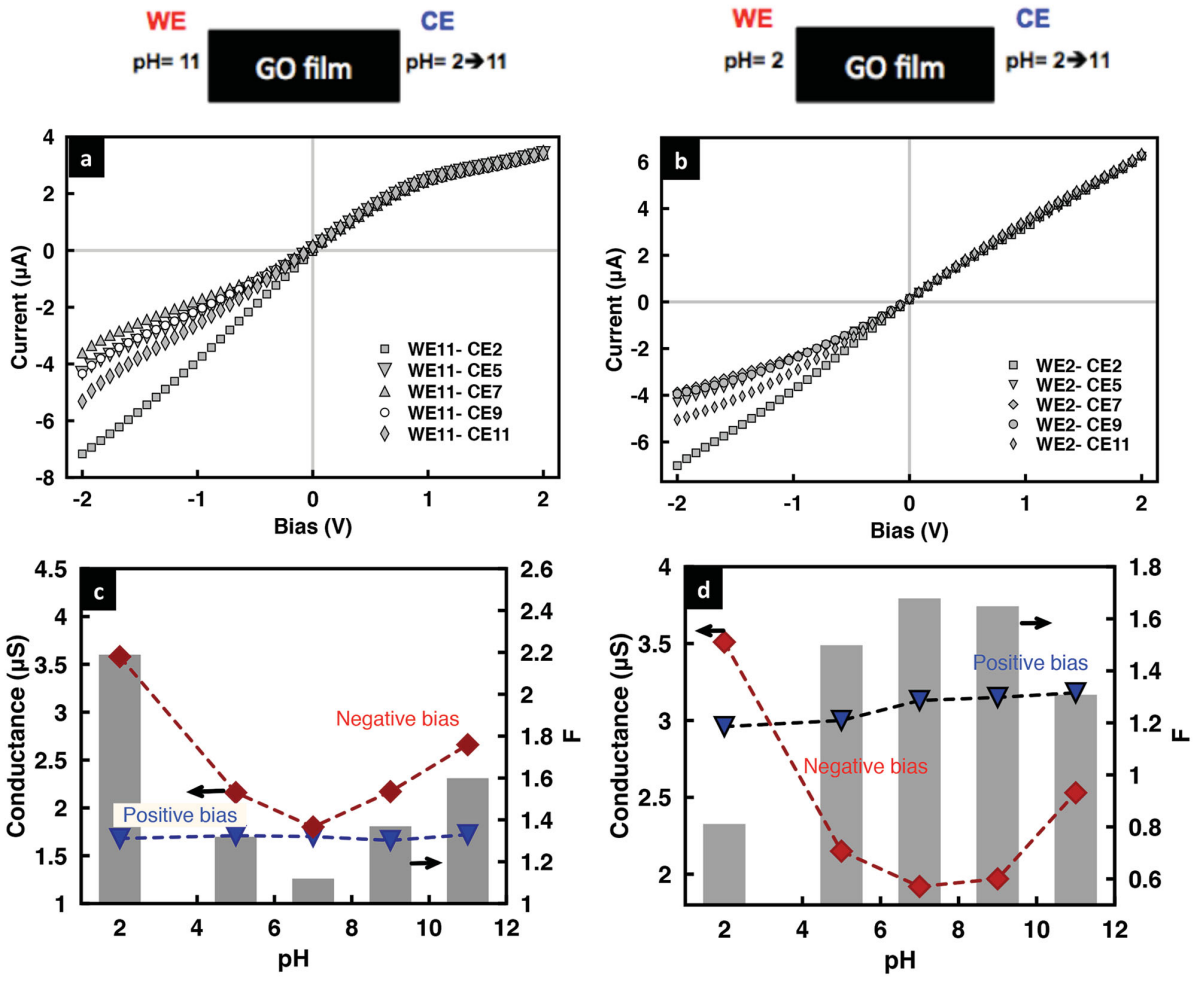
Representative *I*–*V* curves for a geometrically symmetric GO film as the solution pH in the reservoir on either a) the grounded counter electrode (CE) end or b) the working electrode (WE) end is varied while being held constant at the other. c,d) are the corresponding conductance and rectification factor data for both cases, respectively, as a function of the solution pH. The concentration of the KCl solution is 0.1 × 10^−3^ m.

To ascertain the effects of the asymmetry in the solution pH in isolation from that arising from geometrical asymmetry, we repeated these experiments but with a geometrically symmetric film. **Figure** **7** records our observations when the solution pH in the reservoir on the grounded counter electrode (CE) end is similarly ramped from acidic (pH 2) to basic (pH 11) conditions for both cases when the solution pH in the reservoir on the working electrode (WE) end to which a positive or negative bias is applied is held constant either in acidic or basic state. Consistent with our observations in Section 3.1, we observe the permselectivity *η* to increase and hence (*η* + 1)/(*η* − 1) to decrease from acidic to basic conditions, which generally leads to a decrease in the limiting current (Equation (1)). Again, we however do not observe a monotonic decrease but rather an inflexion around pH 7, which can also be attributed to the collapse of the film above the dissociation point discussed above and in Miansari et al.^30^ In keeping with our observations for geometrically asymmetric films, we also observe the reversal in the rectification at low pH, again likely due to proton adsorption under the highly acidic conditions (Figure 7d); we note the reversal is not observed when the conditions in the reservoir at the WE end were basic (Figure 7c).

### 2.3 Solution Concentration Asymmetry

**Figure 8:**
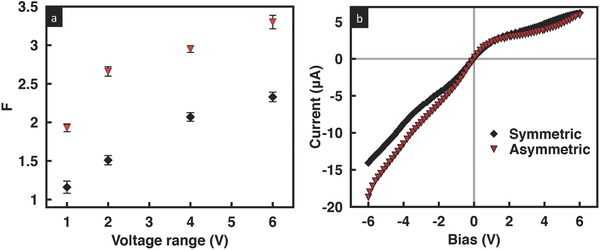
a) Rectification factor and b) representative *I*–*V* curves for a geometrically symmetric film with symmetric electrolyte concentration of 0.1 × 10^−3^ m KCl in the reservoirs at both WE and CE ends (black squares), and an asymmetric electrolyte concentration of 0.1 × 10^−3^ m in the reservoir at the WE end and 10 × 10^−3^ m in the reservoir at the CE end. The pH in both reservoirs is held at 7.

We now turn our attention to investigating the effects of asymmetry obtained by introducing an imbalance in the electrolyte concentration in the anodic and cathodic reservoirs, first for a geometrically symmetric film and subsequently for a geometrically asymmetric one. **Figure** **8**a shows an increase in the degree of rectification in the geometrically symmetric film, as would be expected, when the concentration of the solution on the grounded CE and WE ends were 10 and 0.1 × 10^−3^ m, respectively; the amount by which the rectification increases with the applied voltage, however, remained similar for both cases. The reason for the increase in the rectification with solution concentration asymmetry can be attributed to the increase not only in the limiting current but also in the threshold voltage at which the limiting current for the negative voltage bias occurs (Figure 8b). This is a consequence of a reduction in the Debye length on the CE end as the electrolyte concentration is increased, thus leading to a diminution in the ion permselectivity of the film. The decrease in *η*, corresponding to an increase in (*η* + 1)/(*η* − 1) in Equation (1) then results in an increase in the value for *I*_(−*V*)_. Similarly, the low electrolyte concentration at the WE end leads to a decrease in the limiting current, the corresponding threshold voltage and hence the value of *I*_(+*V*)_ on the positive voltage bias state (Figure 8b). As such, the degree of rectification *F* ≡ |*I*_(−*V*)_/*I*_(+*V*)_| increases with asymmetry in the electrolyte concentration.

**Figure 9:**
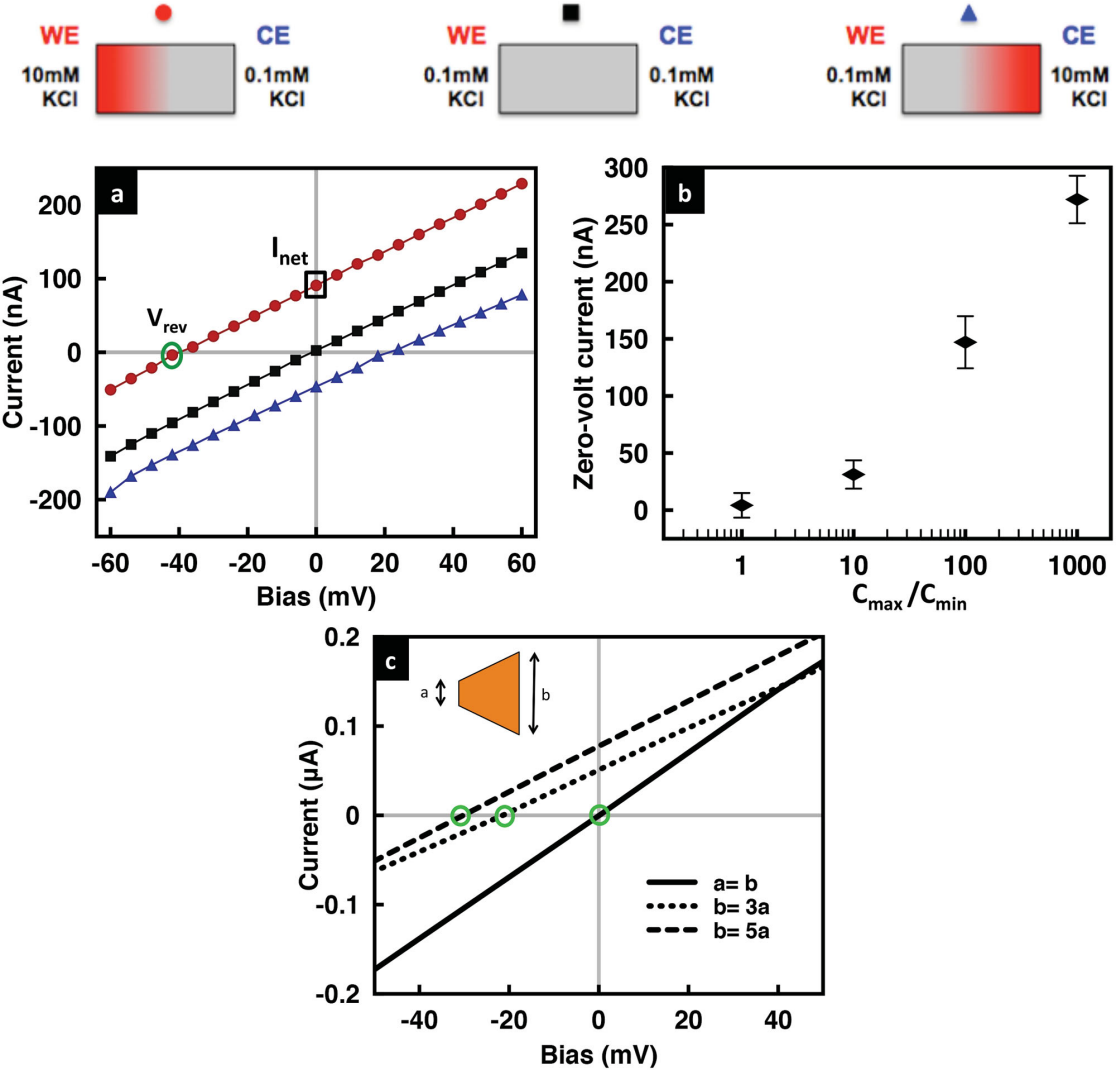
a) *I*–*V* characteristics of a geometrically symmetric film subject to a concentration gradient at pH ≈ 7, showing the existence of a zero‐volt current *I*_net_ (black square along the *y*‐axis) and reversal potential *V*_rev_ (green circle along the *x*‐axis). The KCl concentrations in the reservoirs at the WE and CE ends are shown in the legend above. b) Zero‐volt current as a function of the imposed concentration gradient, characterized by the ratio between the concentration in one reservoir *C*_min_, fixed at 1 × 10^−3^ m, and the concentration in the other *C*_max_, which was increased from 1 × 10^−3^ m to 1 m. c) Corresponding *I*–*V* characteristics of a geometrically asymmetric film with varying ratios of the film cross‐sectional area *b*/*a*, showing the increase in the reversal potential *V*_rev_ (green circles) with increasing geometric asymmetry; the concentration is 1 × 10^−3^ m at both ends.

Further, it can be seen from the low mV *I*–*V* characteristics in **Figure** **9**a that the asymmetric electrolyte concentration across the reservoirs gives rise to a finite current *I*_net_ = *I*_*p*_ +*I*_*n*_, wherein *I*_*p*_ and *I*_*n*_ are the ionic current contributions by the cations (K^+^) and anions (Cl^−^), respectively, even in the absence of an applied potential. Figure 9b shows the dependence of this zero‐volt current that is generated due to net ion diffusion through the interstitial spaces between the sheets that make up the GO film^46^, ^47^ on the difference in the concentration in both reservoirs *C*_max_/*C*_min_; the current was recorded by maintaining the low concentration end at 1 × 10^−3^ m while varying the concentration on the other end from 1 × 10^−3^ m to 1 m. We observe the current to increase drastically with increasing salinity difference. Likewise, the faster cation transport compared to that for anions in the cation‐selective nanochannels comprising the GO film—a consequence of its native negative surface charge and overlapping Debye layers^30^—gives rise to more cation accumulation on one end relative to the other end of the nanochannel, thus producing a potential at zero current, known as the reversal potential^48^, ^49^ *V*_rev_ (Figure 9a). The reversal potential is also present for a geometrically asymmetric film. As seen in Figure 9c, *V*_rev_ increases with increasing geometrical asymmetry, i.e., increasing *b*/*a* values, due to the accumulation of more cations along the wide end of the GO film compared to that at the narrow end such that one end of the film is more positively charged relative to the other, thus producing a larger reversal potential. These observations are, to our best knowledge, the first time electrical power generation has been shown in planar graphene‐based nanofluidic devices simply through the introduction of a salinity gradient across the film, therefore offering an alternative to the membranes used in pressure‐retarded osmosis and reverse dialysis.^47^, ^50^, ^51^

**Figure 10:**
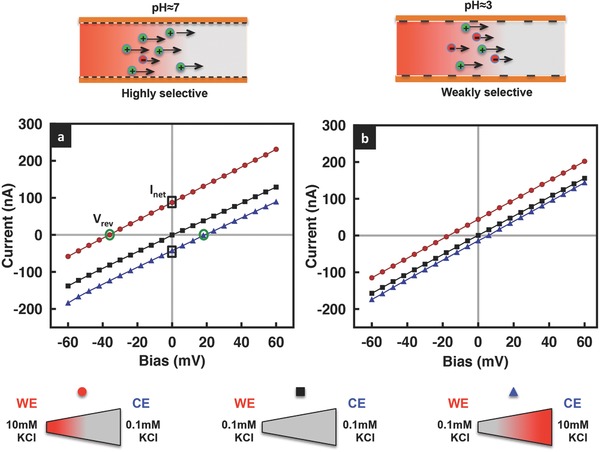
Low voltage *I*–*V* characteristics for a geometrically asymmetric film (*b*/*a* = 3) at a) pH = 7 and b) pH = 3 solutions in which different KCl concentrations are imposed across the reservoirs at the WE and CE ends, as shown by the legend below the plots. The zero‐volt currents *I*_net_ for the different cases are represented by the black squares along the *y*‐axis and the reversal potential *V*_rev_ by the green circles along the *x*‐axis.

**Figure 11:**
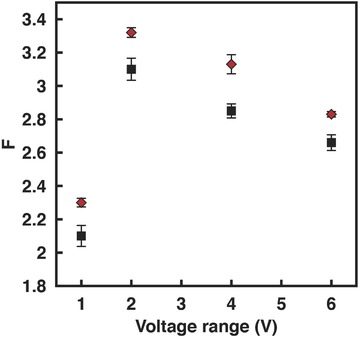
Rectification factor *F* for a geometrically asymmetric film (*b*/*a* = 3) under both symmetric (black squares; 0.1 × 10^−3^ m in both reservoirs) and asymmetric (red diamonds; 0.1 and 10 × 10^−3^ m in the reservoirs at the WE and CE ends, respectively) electrolyte concentrations. The solution pH is maintained at a value of 7.

Further, it can be seen that both *I*_net_ and *V*_rev_ are dependent on the permselectivity of the film and hence the solution pH, whose effect on the permselectivity has already been discussed in Sections 3.1 and 3.2, i.e., the permselectivity of the film increasing with increasing pH. This, as a result, leads to an increase in the net ion flow across the film *I*_net_ as well as the reversal potential *V*_rev_, as seen in **Figure** **10** and **Table** **1**, consistent with that observed elsewhere.^52^ The solution pH is seen to compound the increase in *I*_net_ and *V*_rev_ with larger concentration gradients. In addition to the enhancement in power generation *P*_max_ = *I*_net_ *V*_rev_, we also observe an enhancement in the degree of rectification with increasing concentration gradient, as shown in **Figure** **11**.

**Table 1:** Tabulated values for the zero‐volt current *I*_net_, reversal voltage *V*_rev_, and the power generated *P*_max_ for a geometrically asymmetric film (*b*/*a* = 3) under which different KCl concentrations are imposed in the reservoir at the narrow and wide ends of the film for two different pH values

	Concentration(narrow|wide) [mM|mM]	*I*_net_[nA]	*V*_rev_[mV]	*P*_max_ = *I*_net_ *V*_rev_[nW]
pH ≈ 7	10|1	87.4	36	3.15
	1|1	0	0	0
	1|10	42.7	18	0.77
pH ≈ 3	10|1	44	18	0.79
	1|1	0	0	0
	1|10	14.1	5.99	0.08

## 3 Conclusion

In this work, we have demonstrated ways in which ion current rectification in 2D graphene‐based nanofluidic rectifiers can be enhanced simply by increasing the asymmetry already inherent in the diffusion boundary layer in these systems—shown in recent work to be due to counter‐ion trapping within the sheets that make up the graphene oxide films: by increasing the film's permselectivity through symmetry‐breaking of the surface charge distribution within the film. For example, geometrically asymmetric cuts can be made to the film—a broad 2D analogue of nanofluidic rectifiers comprising conical nanopores; the larger the difference in the cross‐sectional areas between the two ends of the film, the larger the departure from linearity in the current–voltage characteristics of the film owing to the decrease in the limiting current as a consequence of the increased permselectivity. This then leads to an enhancement in the rectification given that the nonlinear behavior occurs only in the positive bias state in which counter‐ion trapping within the film occurs; moreover, the rectification is also observed at lower voltage bias in the asymmetrically cut film. These observations are supported by good agreement obtained between the experimental measurements for the limiting current and that predicted by a simple 1D model for ion transport across a permselective nanochannel membrane.

Additionally, we show the sensitivity of the surface charge distribution and hence the limiting current and rectification factor to the solution pH due to the dissociation of carboxylic ions within the sheets of the graphene oxide films, which increases with decreasing acidity of the solution. Further, we demonstrate that the film's asymmetry and therefore rectification behavior can also be increased by imposing a mismatch between the electrolyte pH or concentration in the reservoirs on both ends of the film. In the latter, the salinity gradient gives rise to the existence of a finite current at zero applied potential, as well as a reversal potential, thus facilitating the possibility for osmotic power generation—the first time this has been demonstrated in these graphene‐based nanofluidic devices to the best of our knowledge.

In summary, these simple ways of enhancing ion current rectification as well as generating electrical power through salinity gradients add to the benefits of low cost, ease, and simplicity of synthesizing, chemically functionalizing, handling, integrating, and scaling up these films for use in practical microdevices for a broad range of applications for biosensing, membrane separation, and energy storage and conversion, among others.

## 4 Experimental Section

Multilayered GO films were prepared by vacuum filtration of a chemically exfoliated GO dispersion (known as the modified Hummers' method^53^, ^54^). To prepare the freestanding film, 100 mL of 0.05 wt% GO solution was first filtered through a cellulose acetate filter membrane (47 mm diameter, 0.2 μm pore size; Whatman Ltd., Maidstone, UK). The GO film was then air‐dried and peeled off from the filter membrane, and cut using a blade into the desirable dimension—in this case, a 3 × 3 mm rectangular geometrically symmetric film and several trapezoidal geometrically asymmetric films of 3 mm widths with varying end lengths. The thickness of the films were measured using a digital micrometer caliper; at least five different locations were measured and the averaged thickness was found to be approximately 30 μm.

**Figure 12:**
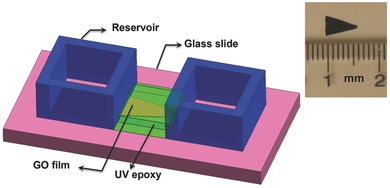
Schematic of the experimental setup comprising two microreservoirs that flank the graphene oxide film mounted onto a glass slide. The working and counter electrodes are immersed in the fluid within the reservoirs. A magnified image of the geometrically asymmetric trapezoidal free‐standing film is shown in the inset.

To ensure that the ion transport occurs solely through the interlayer spacing of the GO film, its top and bottom surfaces were first covered with a layer of UV epoxy; the entire film was then placed on a glass slide and immediately exposed to the UV irradiation. The ends of the film were then connected to two reservoirs glued to both ends of the film, as illustrated in **Figure** **12**. The reservoirs were then filled with potassium chloride (KCl) solutions at different pH and ionic strengths.

The *I*–*V* behavior of the system was observed using a sourcemeter unit (SMU; Model 6430, Keithley Instruments Inc., Cleveland, OH) connected to two Ag/AgCl reference electrodes that were inserted into each half‐cell solution; all measurements were conducted within a Faraday cage. The pH of the solution was adjusted by adding potassium hydroxide (KOH) or hydrochloric acid (HCl). To ensure that the GO film is fully hydrated and swelled before conducting any measurements, both reservoirs were filled with DI water for approximately 2 d prior to their filling with the electrolyte solution at the requisite concentration, after which the system was maintained for a further 12 h. The electrical conductance across the nanochannels was then calculated hourly to obtain the *I*–*V* curves until no detectable changes were observed, i.e., steady‐state was attained while ensuring the film remained fully wetted.

## Acknowledgements

L.Y.Y. acknowledges funding through an Australian Research Council (ARC) Future Fellowship (FT130100672) as well as Discovery Project grant DP140100805. M.M. is also grateful for access to equipment at the Melbourne Centre for Nanofabrication. This work was performed in part at the Melbourne Centre for Nanofabrication (MCN), which is the Victorian Node of the Australian National Fabrication Facility (ANFF).
